# HTLV-1: Regulating the Balance Between Proviral Latency and Reactivation

**DOI:** 10.3389/fmicb.2018.00449

**Published:** 2018-03-19

**Authors:** Anurag Kulkarni, Charles R. M. Bangham

**Affiliations:** Section of Virology, Division of Infectious Diseases, Department of Medicine, Imperial College London, London, United Kingdom

**Keywords:** HTLV-1, latency, virus, metabolism, hypoxia, glucose, epigenetic regulation, transcription

## Abstract

HTLV-1 plus-strand transcription begins with the production of doubly-spliced *tax/rex* transcripts, the levels of which are usually undetectable in freshly isolated peripheral blood mononuclear cells (PBMCs) from HTLV-1-infected individuals. However, the presence of a sustained chronically active cytotoxic T-cell response to HTLV-1 antigens in virtually all HTLV-1-infected individuals, regardless of their proviral load, argues against complete latency of the virus *in vivo*. There is an immediate burst of plus-strand transcription when blood from infected individuals is cultured *ex vivo*. How is the HTLV-1 plus strand silenced in PBMCs? Is it silenced in other anatomical compartments within the host? What reactivates the latent provirus in fresh PBMCs? While plus-strand transcription of the provirus appears to be intermittent, the minus-strand *hbz* transcripts are present in a majority of cells, albeit at low levels. What regulates the difference between the 5′- and 3′-LTR promoter activities and thereby the *tax-hbz* interplay? Finally, T lymphocytes are a migratory population of cells that encounter variable environments in different compartments of the body. Could these micro-environment changes influence the reactivation kinetics of the provirus? In this review we discuss the questions raised above, focusing on the early events leading to HTLV-1 reactivation from latency, and suggest future research directions.

## Introduction

Human T-lymphotropic virus type 1 (HTLV-1), also known as Human T-cell leukemia virus type 1, is a retrovirus that mainly infects CD4+ T-cells *in vivo.* In ∼5–10% of the infected individuals, HTLV-1 infection leads to either an aggressive T-cell malignancy, adult T-cell leukemia/lymphoma (ATL), or a chronic progressive neuro-inflammatory condition called HTLV-1-associated myelopathy/tropical spastic paraparesis (HAM/TSP). Both these conditions have limited treatment options, and ATL in particular carries a very poor prognosis.

Initial infection is asymptomatic and occurs through breast milk, blood, or semen of infected individuals (Bangham and Matsuoka, 2017). HTLV-1 replicates in the host through two distinct routes: (i) Infectious spread: this mode of spread involves productive replication from the integrated provirus followed by the transfer of newly produced virions through the virological synapse (Igakura et al., 2003; Pais-Correia et al., 2010). This is the major route of viral spread in the initial stages of infection when the proviral load – the percentage of infected PBMCs – is low (Bangham et al., 2014). Infectious spread results in the formation of distinct T-cell clones, each clone carrying a single-copy HTLV-1 provirus integrated in a unique genomic location within the host genome. (ii) Mitotic spread: proliferation of HTLV-1-infected host cells results in passive replication of the integrated HTLV-1 provirus within their genome. The two daughter cells resulting from mitosis of an HTLV-1-infected parent cell carry the provirus in the same genomic integration site. In contrast to infectious spread, the contribution of this mode to the proviral load in infected individuals may be small in the early stages of infection but gradually increases during the chronic stage of infection (Bangham et al., 2014). Although the proviral load in each host can fluctuate by a small factor (2- to 5-fold) over time, the proviral loads can vary between infected individuals by over 1000-fold (Nagai et al., 1998; Demontis et al., 2013). Individuals with a higher proviral load are at greater risk of developing either ATL or HAM/TSP (Matsuzaki et al., 2001; Iwanaga et al., 2010). An infected individual typically carries about 10^4^ to 10^5^ different T-cell clones, each with a unique proviral integration site (Bangham et al., 2014). The extent of proliferation of HTLV-1-infected T-cell clones, and thus their respective contribution to an individual’s proviral load, both vary greatly from one clone to another.

**FIGURE 1:**
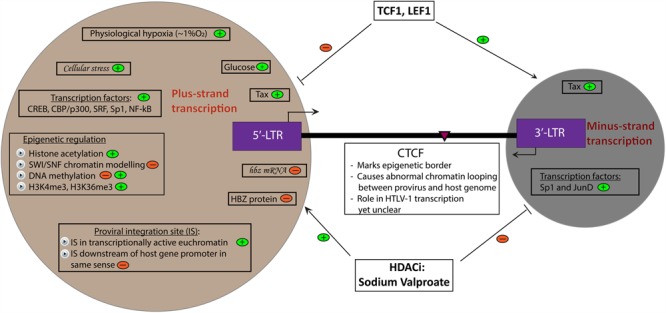
Determinants of HTLV-1 plus- and minus-strand transcription. A summary of currently known factors that induce (depicted by 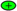) and inhibit (depicted by 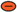) plus- and minus-strand transcription of the HTLV-1 provirus. Italicized words denote hypothetical factors. The pink arrowhead marks the position of the CTCF binding site in the provirus.

A peculiar characteristic of HTLV-1 is the absence of detectable cell-free virions in infected individuals (Demontis et al., 2015). HTLV-1 was previously considered to be latent in infected individuals because there are no detectable plus-strand viral structural RNA or protein products in the peripheral blood mononuclear cells (PBMCs) freshly obtained from HTLV-1 infected individuals. Also, HTLV-1 is genetically stable, with minimal sequence variation over evolutionary time (Gessain et al., 1992), suggesting that *de novo* viral replication, which generates sequence variation, contributes little to the long-term persistence of HTLV-1 *in vivo*. However, a sustained chronically active CTL response to HTLV-1 can be observed in virtually all infected individuals (Jacobson et al., 1990; Bangham, 2009). This observation suggests that the host immune system routinely encounters viral antigens *in vivo*. Since the HTLV-1 plus-strand appears to be latent in peripheral blood, the implication is that plus-strand expression occurs either in intermittent bursts, or outside the circulation, or both. Thus it is necessary to understand the determinants of HTLV-1 latency and reactivation *in vivo* (**Figure 1**) to devise effective preventive and therapeutic approaches against HTLV-1-associated diseases such as HAM/TSP and ATL.

### Proviral Genomic Integration Site

HTLV-1 cannot be looked at in isolation because it is an inseparable part of the chromatinized host genomic DNA. Can we predict the behavior of the integrated provirus in the context of its flanking host genome? HTLV-1 integration favors transcriptionally active regions of the host genome (Melamed et al., 2013). Recently, the host enzyme Protein Phosphatase 2A (PP2A) was identified as a major host co-factor for the HTLV-1 integrase, which could influence the selection of genomic integration sites (Maertens, 2016). Furthermore, plus-strand transcription is silenced when the viral DNA is integrated downstream of a host gene promoter in the same-sense orientation, possibly by transcriptional interference (Melamed et al., 2013). Similarly, the presence of the SWI/SNF-associated ATPase BRG-1 (identified by chromatin immunoprecipitation) upstream of the integrated HTLV-1 provirus is associated with silencing of plus-strand transcription, but its presence downstream of the HTLV-1 sequence is associated with proviral plus-strand expression (Melamed et al., 2013). Recently, it was reported that integration of HTLV-1 in the vicinity of cancer driver genes causes either premature interruption of transcription or antisense-dependent *cis*-perturbation of these genes, perhaps contributing to leukemogenesis (Rosewick et al., 2017).

The discovery of a functional CTCF binding site within the pX region of the HTLV-1 genome adds a new dimension to the importance of the flanking genome to proviral transcription (Satou et al., 2016). CTCF is a critical host protein which binds to an insulator motif in the DNA and appears to limit the spread of epigenetic modifications (Merkenschlager and Nora, 2016). Consistent with this function, the CTCF binding region in HTLV-1 is associated with the presence of a sharp epigenetic border in several histone and DNA modifications. It is possible that the epigenetic border formed by CTCF binding within the HTLV-1 provirus allows unhindered minus-strand transcription while reversibly inhibiting the plus-strand activity. Another central function of CTCF is the formation of chromatin loops, which regulate contacts between enhancers and promoters. It is thus possible that the CTCF bound to the provirus produces abnormal chromatin loops, resulting in aberrant cellular gene expression (Cook et al., 2017). Experiments are now in progress to test these hypotheses. Ultimately, this aberrant gene expression may be an important factor in the development of ATL.

### The Tax-HBZ Interplay

Gene transcription in HTLV-1 progresses in both the sense and anti-sense directions, governed by promoters in the identical long terminal repeats (LTRs) situated at each end of the genome.

#### Plus-Strand Transcription

This is regulated by the TATA-box-containing inducible promoter in the 5′-LTR (Miyazato et al., 2016). TATA boxes are frequently observed in promoters of cellular genes which are highly regulated and responsive to stress and extracellular signals (Basehoar et al., 2004; Bahrami and Drablos, 2016). The 5′-LTR contains three tandem 21-bp imperfect nucleotide repeats called tax-responsive elements (TREs) which bind to the ATF/CREB family of proteins to activate HTLV-1 plus-strand transcription (Armstrong et al., 1993). The transactivator protein Tax, encoded by the pX region of the HTLV-1 genome by a double-splicing mechanism, is the most potent activator of plus-strand transcription and initiates a positive-feedback loop which induces transcription and promotes replication (Beimling and Moelling, 1992; Anderson and Dynan, 1994). Tax enhances the binding of CREB to the TRE, thereby promoting the transactivation of the proviral plus-strand (Suzuki et al., 1993). In addition to ATF/CREB family, the 5′-LTR also has binding sites for several major transcription factors, namely NF-κB, SRF and Sp1, which play direct or indirect roles in activating plus-strand transcription (Armstrong et al., 1993). In addition to Tax, the HTLV-1 pX region encodes another regulatory protein, Rex, which regulates the stabilization of unspliced and singly spliced viral mRNAs, their nuclear export and subsequent effective translation of the viral proteins (Nakano and Watanabe, 2012). The other HTLV-1 pX region plus-strand products play accessory roles *in vivo*: p30 and p13 promote infectivity in a rabbit model (Bartoe et al., 2000; Silverman et al., 2004; Hiraragi et al., 2006), and p30 and p12/p8 enhance persistent infection in macaques (Valeri et al., 2010; Pise-Masison et al., 2014). p13 increases the production of reactive oxygen species, selectively killing transformed cells, and may favor persistence *in vivo* (Silic-Benussi et al., 2010). However, these genes are not essential for viral replication or T-cell immortalization (Derse et al., 1997; Robek et al., 1998), and may not be required for transmission (Furukawa et al., 2004).

#### Minus-Strand Transcription

This is initiated at the 3′-end of the provirus and progresses in the anti-sense direction (Gaudray et al., 2002). It is governed by the TATA-less promoter in the 3′-LTR (Yoshida et al., 2008), which contains three TREs and three Sp1 binding sites (Yoshida et al., 2008; Ma et al., 2016). *sHBZ* is the major transcript driven by the 3′-LTR.

The interplay between the plus-strand and minus-strand transcription is a critical and poorly understood aspect of the HTLV-1 life cycle. We infer that Tax expression *in vivo* is intermittent and highly regulated as it is a very immunogenic protein (Jacobson et al., 1990; Parker et al., 1992). There is no detectable plus-strand gene expression in freshly isolated PBMCs from HTLV-1 infected individuals (Kinoshita et al., 1989; Rende et al., 2011). HBZ, in contrast, is poorly immunogenic and is expressed at a very low level; however, the presence of a detectable cytotoxic T-lymphocyte (CTL) response to HBZ is associated with a lower proviral load (MacNamara et al., 2010). This constant, low-level *hbz* mRNA expression is thought to be necessary for the persistence of HTLV-1 *in vivo*: *hbz* expression can be observed in all HTLV-1-infected individuals, including those with HAM/TSP and ATL (Yoshida et al., 2008). Tax and HBZ have strong effects on a number of cellular processes and signaling cascades (Ma et al., 2016); the effects of the two proteins frequently act in opposition. Certain transcription factors including TCF1 and LEF1 inhibit plus-strand transcription from the 5′-LTR while simultaneously enhancing minus-strand transcription from the 3′-LTR (Ma et al., 2015). In contrast, the histone deacetylase inhibitor (HDACi) valproate activates plus-strand transcription while inhibiting expression of the minus-strand (Lezin et al., 2007; Belrose et al., 2011; Olindo et al., 2011). It is also known that Tax activates minus-strand transcription through binding of the TREs in the 3′-LTR (Landry et al., 2009). Recently, it was shown by single-molecule RNA-FISH that, at a single-cell level, both plus- and minus-strand transcription occurs in bursts, albeit at different times and intensities. HTLV-1 plus-strand mRNA transcription is enhanced in the absence of minus-strand transcripts, while high-level plus-strand transcription promotes transcription from the minus-strand (Billman et al., 2017), generating a putative negative feedback inhibition of plus-strand expression. Until now, it was believed that all HTLV-1-infected cells express *hbz* mRNA at all times; however, this study showed that, in naturally-infected T-cell clones isolated by limiting dilution from HTLV-1-infected individuals, not all HTLV-1-infected cells in each clone are *hbz* positive (Billman et al., 2017) at a given time. Many questions remain unanswered. Most *hbz* transcripts were found in the nuclei of cells, consistent with previous observations (Rende et al., 2011) and with the low level of protein expression of HBZ (Shiohama et al., 2016). HBZ protein inhibits *tax* expression by competing for binding of the transcription co-factor CREB (Gaudray et al., 2002; Lemasson et al., 2007; Clerc et al., 2008). However, since HBZ protein is expressed at such a low level in naturally-infected cells, the importance of this mechanism is uncertain (Shiohama et al., 2016).

These observations raise important questions: does *hbz* RNA also inhibit *tax* expression? What are the factors that regulate minus-strand transcriptional bursts? It has been shown that Sp1 co-operates with JunD to activate minus-strand transcription, but how and when this happens in the context of the cell cycle and viral replication are still unknown (Gazon et al., 2012). Recently, Billman et al. (2017) showed at the single cell level, in naturally-infected HTLV-1 positive T-cell clones, that cells in S and G2/M phase of the cell cycle have elevated *tax* and *hbz* mRNA levels, suggesting that the cell cycle plays a role in regulating HTLV-1 transcription. It is now becoming clear that the results of population-level analyses can mask strong single-cell heterogeneity within the population.

### Epigenetic Regulation of HTLV-1 Transcription

Small-molecule inhibitors targeting epigenetic enzymes have been widely used in HIV latency research. Indeed, reactivation of the latent HIV-1 provirus occurs in response to HDACi [e.g., suberanilohydroxamic acid (SAHA)], DNA methylation inhibitors (e.g., 5-azacytidine) and histone methyltransferase inhibitors (e.g., the EZH1/2 inhibitor GSK343) (Spina et al., 2013; Tripathy et al., 2015). Similarly, in the case of HTLV-1, the HDACi valproate and DNA methyltransferase inhibitor 5-azacytidine have been shown to activate the latent HTLV-1 provirus (Koiwa et al., 2002; Belrose et al., 2011). These observations suggest that epigenetic modulations in the promoters, enhancers and gene bodies of the integrated provirus play an important role in proviral transcription. Cytosine methylation of the 5′-LTR of HTLV-1 is associated with silencing of plus-strand transcription; the degree of this 5′-LTR methylation varies from individual to individual, whereas the 3′-LTR is unmethylated in most individuals (Taniguchi et al., 2005), consistent with the constitutive activation of the promoter in the 3′-LTR. However, it is unclear how dynamic the DNA methylation pattern of the provirus is. It is unlikely that the highly regulated promoter in the 5′-LTR of HTLV-1 would rely primarily on DNA demethylation for its reactivation. Data are needed on the extent of DNA methylation during bursts of gene expression from the plus-strand and minus-strand, and whether this methylation is a cause or an effect of promoter activation. Activation of plus-strand transcription is associated with Tax-mediated recruitment of pCREB and CBP/p300, resulting in histone acetylation and nucleosomal remodeling through BRG-1-containing SWI/SNF complexes (Sharma and Nyborg, 2008; Easley et al., 2010). Similarly, HTLV-1 plus-strand transcription is associated with a significant increase in the activatory histone epigenetic modifications H3K4me3 and H3K36me3 in different regions of the HTLV-1 provirus (Kulkarni et al., 2017). It is likely that, as in host gene expression, many epigenetic modifications are a cause as well as consequence of viral reactivation. Most of the epigenetic studies carried out so far have been in *in vitro* cellular models where Tax expression is the primary mechanism of 5′-LTR activation through a positive feedback loop. However, evidence from freshly isolated PBMCs from HTLV-1-infected individuals indicates that there is little or no Tax protein expression *in vivo* at steady state. Hence, it is important to examine the earliest epigenetic changes that occur at the provirus in freshly isolated PBMCs from HTLV-1-infected individuals to provide clues to the changes that occur *in vivo*. It is still unclear what primarily reactivates plus-strand transcription in *ex vivo* PBMCs from HTLV-1-infected individuals. The answer to this conundrum may elucidate the key question – what regulates the rapidly reversible silencing of plus-strand transcription *in vivo*?

### Metabolic Regulation of HTLV-1 Transcription

The metabolic and functional activities of circulating PBMCs depend directly on their micro-environment. What effects do metabolic changes have on the integrated HTLV-1 provirus within these cells? We recently showed that HTLV-1 reactivation from latency is severely limited in the absence of glucose in the surrounding medium (Kulkarni et al., 2017). In support of this conclusion, we found that PBMCs treated with glycolysis inhibitors – and to a lesser extent with mitochondrial electron transport chain (ETC) inhibitors – show a significant impairment in HTLV-1 plus-strand transcription when they are cultured *ex vivo*. The importance of glucose in the HTLV-1 life cycle is emphasized by the fact that the glucose receptor GLUT-1 is a cellular receptor for HTLV-1 (Manel et al., 2003). Surprisingly, drugs that inhibit or stimulate the TCA cycle do not alter HTLV-1 transcription (Kulkarni et al., 2017). Indeed, it is known that different subsets of T-cells use distinct metabolic pathways to satisfy their energy requirements (Dimeloe et al., 2017). How glucose metabolism influences HTLV-1 reactivation from latency is not known. A plausible explanation is that inhibition of glycolysis or the mitochondrial ETC reduces the availability of Acetyl CoA and ATP. Acetyl CoA is an important acetyl group donor in the process of histone acetylation, which is associated with transcriptional activation. Similarly, the SWI/SNF family of chromatin-remodeling complexes such as BAF and PBAF rely on energy in the form of ATP for their nucleosomal remodeling function. It has been shown before that HTLV-1 plus-strand activation is intimately linked with histone acetylation and chromatin remodeling at the 5′-LTR promoter (Easley et al., 2010; Nyborg et al., 2010). Another important question that arises from these results is the possible impact of high blood glucose levels on HTLV-1 replication. Do HTLV-1-infected individuals with poorly controlled diabetes have a higher proviral load of HTLV-1, resulting in a significantly higher risk of HTLV-1-associated diseases such as HAM/TSP or ATL? Further work is needed to clarify the cause and effect relationships between metabolic activity and proviral activation in HTLV-1-infected primary CD4^+^ T-cells.

### Anatomical Regulation of HTLV-1 Transcription

Lymphocytes are constantly exposed to highly variable stresses during circulation through different body compartments. As mentioned above, HTLV-1 appears to be latent in freshly isolated PBMCs from the peripheral blood of HTLV-1 infected individuals. The presence of a chronically active CTL response to HTLV-1 in all infected individuals raises the possibility that HTLV-1 expression is more frequent in different compartments like the bone marrow, lymph, and lymph nodes (Bangham, 2008). Also, Yasunaga et al. (2016) found that *tax* transcription in the bone marrow of STLV-1-infected Japanese macaques was significantly higher than that in other tissues. One factor that differs significantly between these compartments is the oxygen tension. While the lymph, lymph nodes, and bone marrow are highly hypoxic (∼1% oxygen), PBMCs in peripheral venous blood are exposed to significantly higher levels of oxygen (∼6–10%) (Tsai et al., 2004; Hangai-Hoger et al., 2007). We recently reported that, in primary PBMCs isolated from HTLV-1 infected individuals, physiological hypoxia (∼1% oxygen) enhances plus-strand HTLV-1 transcription (Kulkarni et al., 2017). Again, further work is needed to identify the molecular mechanism of this effect, and the consequences of this observation on the establishment and spread of viral infection within and between individuals.

## Summary

The transcriptional regulation of the plus- and minus-strands of HTLV-1 is affected by a number of virus-specific and host-specific factors. Advances in single-cell genomics and transcriptomics have made it possible to study HTLV-1 infection at the single-cell level. We anticipate that these approaches will shed light on the effect of cellular heterogeneity on viral replication and help identify novel mechanisms that were missed by earlier studies which depended on population-level analyses. Precise identification of the molecular mechanisms involved in HTLV-1 reactivation from latency would help us design and test novel therapeutic strategies to reduce the disease burden associated with this infection.

## Author Contributions

Both authors have made a substantial, direct and intellectual contribution to the work, and approved it for publication.

## Conflict of Interest Statement

The authors declare that the research was conducted in the absence of any commercial or financial relationships that could be construed as a potential conflict of interest.
